# TNF is a key cytokine mediating neutrophil cytotoxic activity in breast cancer patients

**DOI:** 10.1038/npjbcancer.2016.9

**Published:** 2016-04-20

**Authors:** Elizabeth Comen, Paulina Wojnarowicz, Venkatraman E Seshan, Riddhi Shah, Courtney Coker, Larry Norton, Robert Benezra

**Affiliations:** 1 Department of Medicine, Memorial Sloan Kettering Cancer Center, New York, NY, USA; 2 Department of Cancer Biology and Genetics, Memorial Sloan Kettering Cancer Center, New York, NY, USA; 3 Department of Epidemiology and Biostatistics, Memorial Sloan Kettering Cancer Center, New York, NY, USA

## Abstract

We have previously shown a novel antimetastatic role for neutrophils in the premetastatic lung of mice in models of breast cancer. Here we expand on those findings in the context of human breast cancer. We assessed the cytotoxicity of neutrophils from 90 newly diagnosed breast cancer patients, 24 ductal carcinoma *in situ* patients, 56 metastatic breast cancer patients, and 64 women with no history of cancer. We report that neutrophils from metastatic and newly diagnosed breast cancer patients are significantly more cytotoxic than neutrophils from cancer-free individuals. We hypothesized that tumor-secreted factors ‘prime’ neutrophils to become cytotoxic. To identify these factors we assayed for cytokines in serum from 54 breast cancer patients and 35 cancer-free controls. Tumor necrosis factor (TNFα), MCP-1 (CCL2), and IL1RA significantly correlated with cytotoxicity and directly stimulated neutrophil cytotoxicity *ex vivo*. RNA-seq analyses found protein kinase C iota (*PRKCI)* to be over expressed in patient neutrophils relative to neutrophils from cancer-free individuals. *PRKCI* has been implicated in NADPH oxidase assembly, required for neutrophil-mediated cell cytotoxicity. Treatment of human neutrophils with TNF-induced *PRKCI* expression and cytotoxicity in samples that had low basal levels of PRKCI expression. To date, this work is the first to demonstrate the cytotoxic role of neutrophils in the peripheral blood of a large cohort of breast cancer patients, and that select cytokines appear to mediate the stimulation of neutrophil cytotoxicity. Further functional studies are necessary to identify clinically relevant means of stimulating neutrophil cytotoxicity as an effective barrier against disease progression and metastasis.

## Introduction

Models of cancer progression have long focused on cell-autonomous aberrations of cancer cells, such as sustained proliferation, resistance to apoptotic signals, and increased invasive characteristics (reviewed in ref. 1). More recently, experimental models suggest that the innate immune system appears essential to tumor progression, working in concert with cancer cells to either inhibit or promote tumor growth and metastasis (reviewed in ref. 2). For example, a primary tumor can modulate the metastatic potential of distant organs by influencing native stromal cells, such as white blood cells, at the premetastatic site.^3,4^

Neutrophils make up between 40 and 75% of the white blood cells in circulation and are traditionally associated with innate defence against infection; however, several recent studies have implicated neutrophils in cancer development and progression. Accumulating evidence suggests that neutrophils may have a dual role in cancer—acting in a pro-tumorigenic manner in some contexts^4,5^ and displaying anti-tumorigenic functions in others.^6^ One hypothesis is that the polarization of neutrophils, as either pro- or anti-tumorigenic agents in the cancer setting reflects differences in the relative microenvironment, resulting in neutrophils in the circulation functioning differently from those localized to the tumor site.^6^ Preliminary experimental work indicates that select cytokines including but not limited to CCL2, CCL3, CCL4, CCL8, CCL12, CCL17, CCL20, CXCL5, tumor necrosis factor (TNFα), transforming growth factor (TGF)-β, and interleukin (IL)-8 modulate neutrophil function and may contribute to neutrophil polarization (reviewed in ref. 7). In addition to the influence of the microenvironment, neutrophil function may be related to the stage of disease. Acharyya *et al.*^5^ demonstrated that neutrophils from mice bearing established tumors and metastases secreted the prosurvival factors S100A8/9, which promoted breast cancer cell survival and the development of additional metastases. In contrast, in early stage, premetastatic disease, Granot *et al.*^6^ demonstrated in mouse models that select neutrophils are mobilized and entrained by a primary breast tumor to become cytotoxic against cancer cells; this suggests that neutrophils may acquire the capacity to inhibit metastatic seeding in the lung. Furthermore, recent evidence suggests that the oncogenic context of a tumor may also have a role in determining neutrophil polarization. Finisguerra *et al.*^8^ showed that MET signaling, while promoting cancer cell proliferation and survival, is also required for the recruitment and cytotoxic activity of anti-tumoral neutrophils.

Translating these findings to human disease, we previously demonstrated that neutrophils collected from the peripheral blood of women newly diagnosed with primary breast cancer were cytotoxic to breast cancer cell lines *ex vivo*. In contrast, neutrophils from healthy female controls, with no history of cancer, were significantly less cytotoxic to breast cancer cell lines.^6^ Although experimental data demonstrates that select cytokines modulate neutrophil function, little is known about the function of circulating cytokines in altering neutrophil phenotypes, such as anticancer cell cytotoxicity, in cancer patients. Clinical practice assumes that neutrophils function similarly in breast cancer patients as they do in healthy women. For example, clinicians routinely boost neutrophil counts so as to limit chemotherapy toxicity and enable treatment delivery.

In the present study, we sought to evaluate patterns of cytokine expression in peripheral blood collected from healthy women and breast cancer patients and related these levels to the cytotoxicity of neutrophils isolated from the same blood samples. We hypothesized that neutrophils from the peripheral blood of newly diagnosed breast cancer patients, as compared with healthy controls, are exposed to different cytokines and that these cytokines modulate differences in neutrophil cytotoxicity. To identify these factors we preformed a discovery ELISA-based screen testing for cytokines in the serum of patients and controls and determined neutrophil cytotoxicity in co-culture experiments in the same samples. We also carried out RNA-sequencing (RNA-seq) on a subset of these neutrophil samples to investigate alterations in expression profiles. We demonstrate that circulating neutrophils from breast cancer patients have significantly increased anticancer cell cytotoxicity and that this activity can be stimulated by the cytokine TNF (TNFα). *PRKCI* is elevated in cytotoxic neutrophils and can be stimulated by TNF in neutrophils with low basal levels of *PRKCI*. Our findings suggest that the cytokine profiles of breast cancer patients may be indicative of the cytotoxicity of the patient’s neutrophils, and raise the possibility of novel immunotherapies whereby patient neutrophils are treated with select cytokines to stimulate anticancer cell cytotoxicity, both of which may prove helpful for the clinical management of breast cancer patients.

## Results

### Cytotoxicity of neutrophils from breast cancer patients and controls

**Figure 1:**
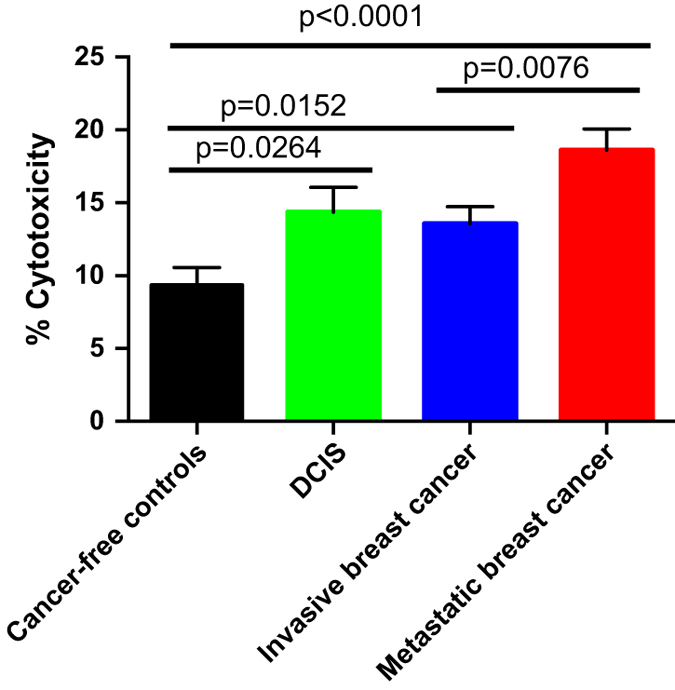
Cytotoxicity of neutrophils from cancer-free controls, patients with DCIS, invasive and metastatic breast cancer. Blood was drawn from 64 cancer-free controls, 87 patients with primary non-metastatic breast cancer, 22 with DCIS, and 56 with metastatic breast cancer and neutrophils were isolated. Neutrophils were co-cultured overnight with the luciferase expressing breast cancer cell line MDA-MB-231. Following co-culture, luciferase activity was determined as readout for cell viability, and percent cytotoxicity (decrease of cell viability) was calculated relative to MDA-MB-231 cell monocultures. Mean cytotoxicities are shown for cancer-free controls, DCIS patients, non-metastatic invasive breast cancer and metastatic breast cancer patients. DCIS, ductal carcinoma *in situ*.

In our previous study, investigating the role of neutrophils in mouse models of breast cancer metastasis, it was observed that neutrophils from tumor-bearing mice displayed higher cytotoxic activity against breast cancer cells relative to neutrophils from cancer-free control mice. Here we wanted to further explore this cytotoxic phenotype in the context of human breast cancer by analyzing neutrophils isolated from cancer-free individuals and breast cancer patients, including various clinical subtypes and disease states. In this effort we collected blood from 64 cancer-free controls, 90 women with primary, untreated, non-metastatic breast cancer, 24 women with ductal carcinoma *in situ* (DCIS), and 56 women with metastatic disease. Neutrophils were isolated from these freshly drawn blood samples, and co-cultured overnight with the luciferase expressing human breast cancer cell line MDA-MB-231. After overnight co-culture, luciferase activity was assayed as a reflection of cancer cell viability. Consistent with the observations of Granot *et al.*, we found that neutrophils from patients with primary invasive breast cancer were significantly more cytotoxic against MDA-MB-231 cells relative to cancer-free controls (13.6% vs. 9.4%, *P*=0.0152; Figure 1). Neutrophils from patients with DCIS also had higher cytotoxicity relative to cancer-free controls (14.4% vs. 9.4%, *P*=0.0264; Figure 1). Furthermore, neutrophils isolated from women with metastatic breast cancer displayed even higher cytotoxic activity (18.6%) relative to both cancer-free controls and patients with primary breast cancer (*P*<0.0001 and *P*=0.0076, respectively; Figure 1). We also divided cancer samples according to their hormone status, tumor stage, and lymph node involvement, and observed trends towards higher neutrophil cytotoxic activity in samples with HER2− status, earlier stage and low lymph node involvement (Table 1; Supplementary Table 1; Supplementary Table 2). However, the sample numbers were too small to assess the significance of these observations.

### Serum profiling of breast cancer patients and controls

**Figure 2:**
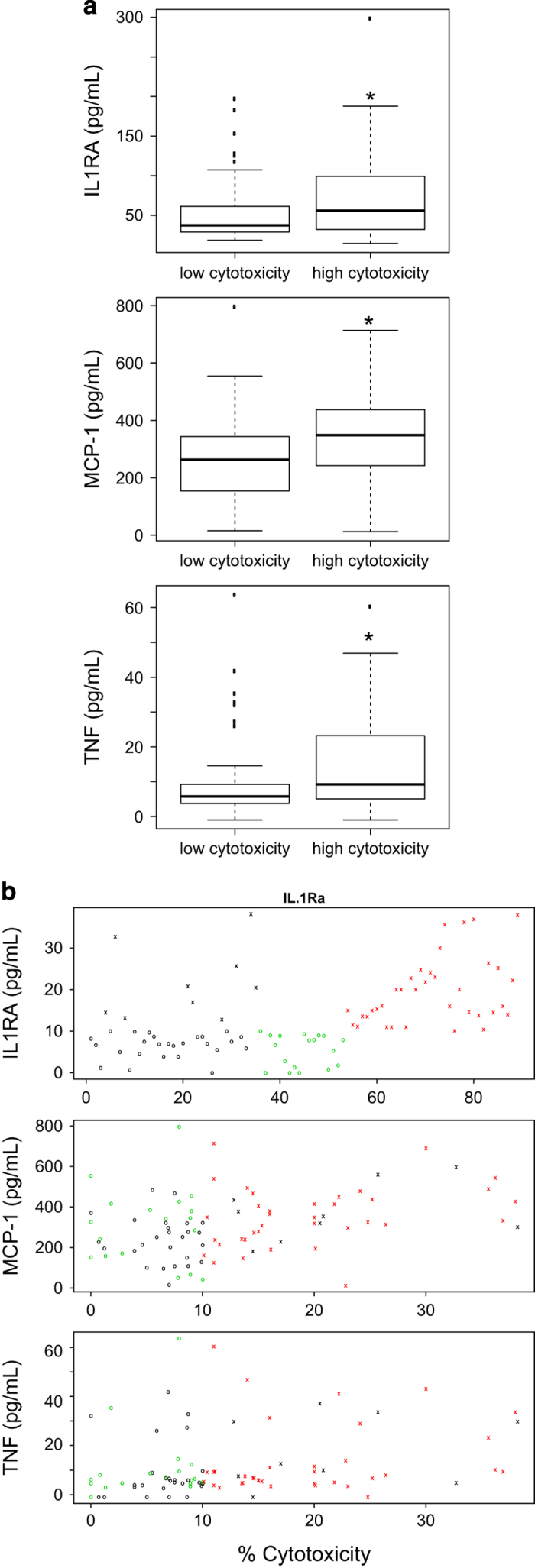
MCP-1, IL1RA, and TNF profiling in serum samples from cancer-free controls and patients with primary breast cancer. (**a**) Box plots showing the mean cytokine level (pg/ml) in samples with high and low neutrophil cytotoxicity. (**b**) Scatter plots showing correlations between cytokine level and cytotoxicity (percent cytotoxicity) of neutrophils from cancer-free controls (*n*=35) (black circles and crosses), primary breast cancer patients with low neutrophil cytotoxicity (*n*=18; green circles) and high neutrophil cytotoxicity (*n*=36; red crosses). Significant differences are indicated with *P<0.05. IL, interleukin; TNF, tumor necrosis factor.

The observation that circulating neutrophils from breast cancer patients show enhanced cytotoxicity relative to cancer-free controls, and the finding that the primary tumor ‘entrains’ the cytotoxic activity observed in circulating neutrophils in mouse models,^6^ suggest that there may be molecular signals released by the primary tumor that stimulate neutrophil cytotoxicity. To address this hypothesis, we performed a pilot exploratory study to profile cytokines in human serum samples from a subset of the samples for which neutrophil cytotoxicity data had been collected. Serum profiling was performed using a Milliplex Cytokine/Chemokine multiplex assay, which evaluated the levels of 39 cytokines in 15 cancer-free controls, 11 patients with high neutrophil cytotoxicity, and 11 patients with low neutrophil cytotoxicity. In our analyses, three cytokines, MCP-1 (CCL2), IL1A, and TNF, were present at significantly higher concentrations in patients relative to controls (data not shown). We then sought to evaluate these three cytokines (plus IL1RA) in a larger sample size of 89 independent serum samples. The samples included 54 breast cancer patients and 35 cancer-free controls. We found IL1RA, MCP-1, and TNF to be significantly correlated with cytotoxicity (*P*=0.016, *P*=0.003, and *P*=0.005, respectively), and present at significantly higher levels in samples with high neutrophil cytotoxicity relative to samples with low neutrophil cytotoxicity (*P*<0.05; Figure 2).

We also performed cytokine profiling for IL1A, IL1RA, MCP-1, and TNF on the serum obtained from 55 of the 56 metastatic breast cancer patients. Within the metastatic sample group, the level of cytokine present in the serum did not correlate with cytotoxicity for any of the cytokines tested, which may be due to the saturating high levels of cytokines within the population (Supplementary Figure 1). To bolster this claim, we performed cytokine profiling on a subset of 13 samples from women with no history of cancer, 13 samples from patients with primary breast cancers with no evidence of metastasis, and 13 samples from metastatic patients on the same array. (We were not able to compare the values from metastatic serum samples to those of controls or patients with primary breast cancer that were performed at different times on different arrays). MCP-1 and TNF were present at the highest concentrations in serum from metastatic patients relative to the other populations (*P*=0.038 and *P*=0.0053; Supplementary Figure 1).

### Circulating cytokines stimulate neutrophil cytotoxicity

**Figure 3:**
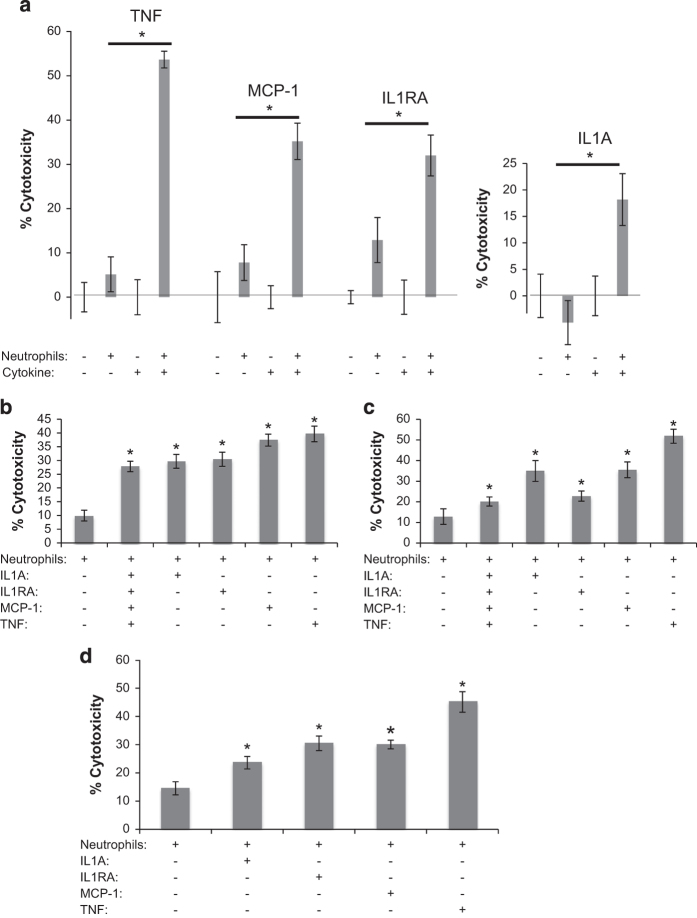
*In vitro* stimulation of neutrophil cytotoxicity. (**a**) Freshly isolated neutrophils from cancer-free controls were co-cultured overnight with MDA-MB-231 cells either in the presence or absence of the indicated cytokines. Freshly isolated neutrophils were incubated with the indicated cytokines for 2 h, after which cytokine was washed out and neutrophils were co-cultured overnight with MDA-MB-231 cells, with results for neutrophils isolated from cancer-free controls, patients with primary breast cancer and patients with metastatic breast cancer are shown in (**b**–**d**), respectively. Following overnight co-culture luciferase activity was determined, and percent loss of luciferase activity relative to untreated MDA-MB-231 (or MDA-MB-231 in the presence of the indicated cytokine for (**a**)) was calculated as a readout for percent cytotoxicity. MCP-1 (CCL2), IL1RA, IL1A, and TNF were administered at 100 ng/ml. Significant differences are indicated with *P<0.05. IL, interleukin; TNF, tumor necrosis factor.

The study by Granot *et al.* previously demonstrated that select chemokines were able to stimulate cytotoxic activity in neutrophils derived from mice. We tested whether IL1A, MCP-1, IL1RA, and TNF were capable of stimulating neutrophil cytotoxicity to determine whether the increased serum levels of these cytokines in breast cancer patients were directly related to the cytotoxic activity of neutrophils. We found that MCP-1, IL1RA, IL1A, and TNF were capable of significantly stimulating neutrophil cytotoxicity relative to unstimulated neutrophils after overnight co-culture, with TNF showing the greatest induction of neutrophil cytotoxic activity (Figure 3a). Furthermore, we found that incubation of neutrophils, freshly isolated from cancer-free controls, with the cytokines for 2 h, followed by washing out the cytokine and incubating these neutrophils with MDA-MB-231 cells overnight was also able to stimulate this cytotoxic activity. Thus, short-term incubation with a stimulating cytokine is sufficient to stimulate the cytotoxic activity of neutrophils isolated from cancer-free controls (Figure 3b).

We then tested whether these cytokines were capable of further stimulating neutrophil cytotoxicity in neutrophils isolated from breast cancer patients. As was observed with neutrophils from cancer-free controls, MCP-1, IL1RA, IL1A, and TNF were capable of significantly enhancing the cytotoxic activity of neutrophils from breast cancer patients (Figure 3c). These cytokines also significantly enhanced the cytotoxic activity of neutrophils from metastatic breast cancer patients (Figure 3d). Thus, the cytotoxicity of neutrophils from patients, which on average have higher baseline cytotoxicity, could be further enhanced with the identified stimulatory cytokines to boost the baseline cytotoxicity of these tumor-entrained neutrophils. Interestingly, there did not appear to be a synergistic effect on neutrophil cytotoxic activity from incubating the neutrophils with all four stimulatory cytokines at once, and TNF showed the greatest stimulatory effect in 9 of 11 (82%) neutrophil samples tested with multiple cytokines (data not shown).

### RNA-seq analysis of neutrophils

**Figure 4:**
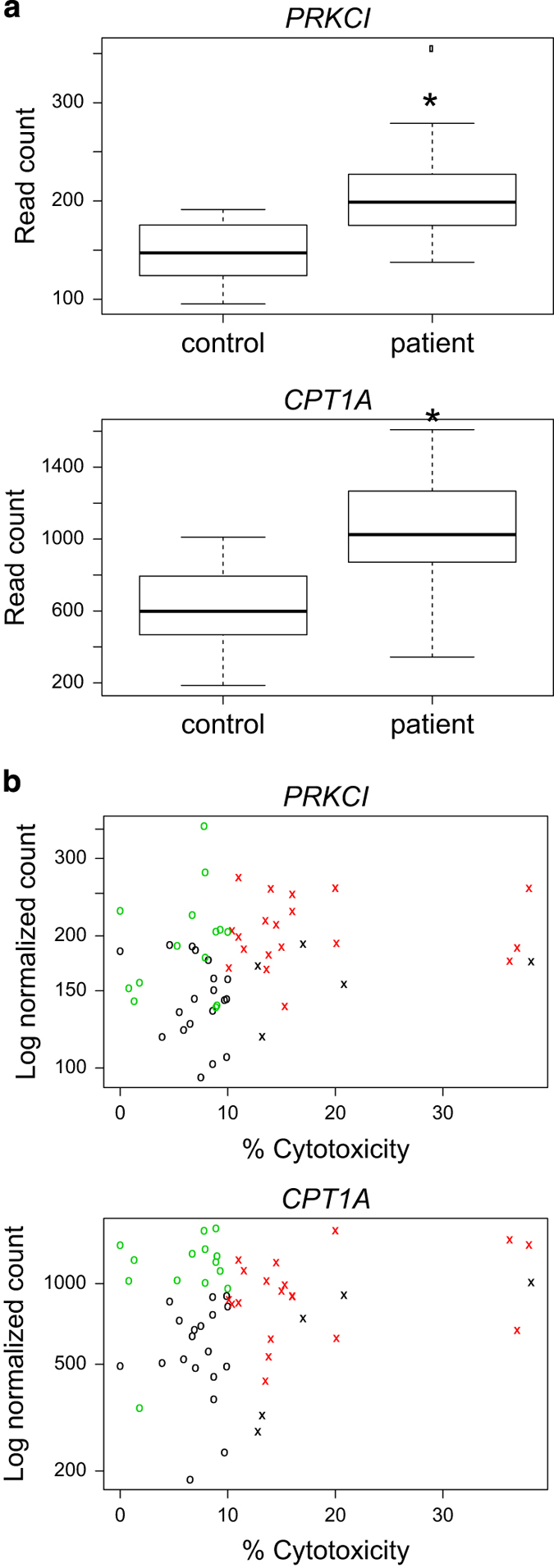
RNA-seq analyses of neutrophils from cancer-free controls and patients with primary breast cancer. (**a**) Box plots showing gene expression of *PRKCI* and *CPT1A*, with samples grouped as cancer-free controls (*n*=25) or breast cancer patients (*n*=35). (**b**) Scatter plots showing correlations between gene expression level and cytotoxicity (percent cytotoxicity) of neutrophils from cancer-free controls (black circles and crosses), breast cancer patients with low neutrophil cytotoxicity (*n*=14; green circles) and high neutrophil cytotoxicity (*n*=21; red crosses). Significant differences are indicated with *P<0.085.

To further investigate the features of the circulating neutrophils from breast cancer patients and controls, we performed RNA-seq analyses to identify genes that are associated with neutrophil cytotoxicity. Of the 89 samples that we had previously profiled for serum cytokine concentrations and neutrophil cytotoxicity, 60 samples had neutrophil RNA suitable for RNA-seq analysis. These 60 samples included 25 controls, and 35 patients (14 with low neutrophil cytotoxicity and 21 with high cytotoxicity). Differential gene expression analysis using a Wilcoxon–Mann–Whitney rank sum, with Benjamini–Hochberg false discovery rate correction (<10%), identified two genes that were differentially expressed between patients and controls. These genes were *PRKCI* and *CPT1A* (*P*=0.058 and *P*=0.014, respectively; Figure 4). *PRKCI* had a Spearman correlation of 0.29, and an unadjusted *P*-value of 0.027, suggesting a positive correlation of *PRKCI* gene expression level and neutrophil cytotoxic activity.

### *PRKCI* gene expression induction by TNF

**Figure 5:**
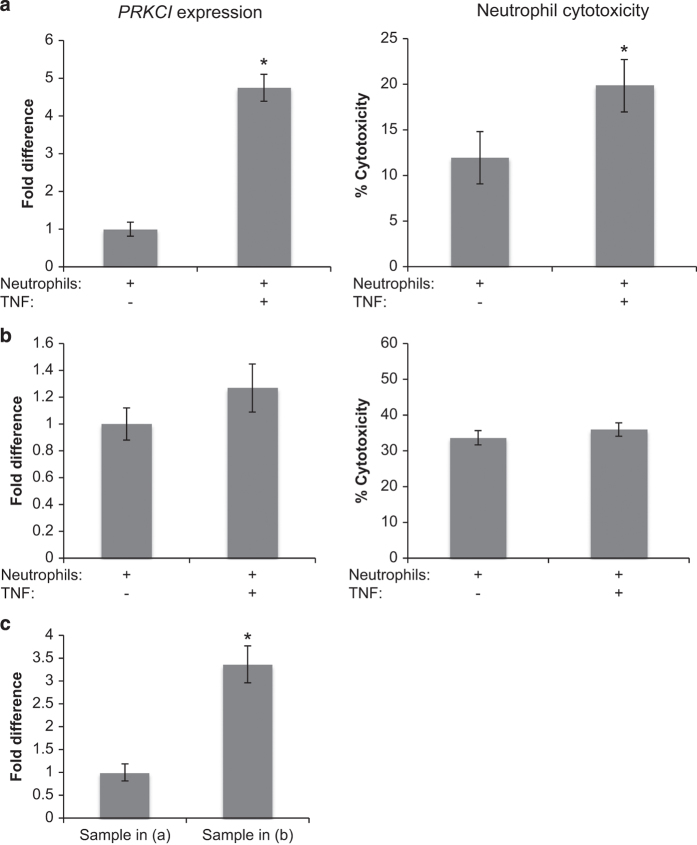
*PRKCI* expression in cytokine-stimulated neutrophils. Left panels, *PRKCI* expression, by qRT-PCR, in neutrophils following 2 h of incubation with TNF (100 ng/ml). Right panels, neutrophil cytotoxicity of the corresponding neutrophil samples following washout of the cytokine and overnight co-culture with the MDA-MB-231 cell line. (**a**) Example of *PRKCI* upregulation and stimulation of cytotoxic activity in response to TNF treatment in the same sample. (**b**) Example of no *PRKCI* upregulation or stimulation of cytotoxic activity in response to TNF in the same sample. (**c**) Relative levels of endogenous *PRKCI* expression in the samples shown in (**a**) and (**b**). Significant differences are indicated with *P<0.05. TNF, tumor necrosis factor.

To validate the findings of our RNA-seq analysis, we performed a quantitative reverse transcription PCR (qRT-PCR) analysis on a subset of neutrophil samples. Of the two protein-coding genes identified as differentially expressed between patients and controls, only *PRKCI* expression was detectable by qRT-PCR in neutrophil samples. To investigate whether the elevated levels of *PRKCI* in patients relative to controls are related to TNF and cytotoxicity, both of which are also on average elevated in patients relative to controls, we treated neutrophil samples from cancer-free controls with TNF and assayed for *PRCKI* expression by qRT-PCR. In four neutrophil samples we found that 2 h of TNF incubation led to an upregulation of *PRKCI* (one representative sample is shown in Figure 5a, left panel). This upregulation correlated with increased neutrophil cytotoxicity against MDA-MB-231 cells (Figure 5a, right panel). In another four neutrophil samples, TNF treatment did not result in upregulation of *PRKCI* (one representative sample is shown in Figure 5b, left panel), however, the baseline cytotoxicity was already relatively high in these samples (Figure 5b, right panel). Furthermore, the basal level of *PRKCI* expression of the sample shown in Figure 5b was significantly higher than the sample shown in Figure 5a (Figure 5c). These results suggest that *PRKCI* expression is correlated with cytotoxicity, and can be induced by TNF in a subset of neutrophils that have relatively low basal levels of *PRKCI* expression. Our data also suggest that neutrophils with higher levels of *PRKCI* may exhibit increased cytotoxic activity, and in such cases TNF stimulation may be redundant.

## Discussion

In the last few decades, while breast cancer mortality rates have decreased, metastatic spread continues to drive mortality rates. Over the past decade attention has turned to the tumor microenvironment, circulating modifiers of metastatic disease, and how the immune system may regulate tumor growth and progression. In the present study, we present three key findings reflecting the potential role of neutrophils in mediating cancer progression. First, in an expanded, and more clinically diverse patient population, we reproduce and validate our initial findings that select circulating neutrophils in breast cancer patients are cytotoxic to breast cancer cells when compared with those obtained from healthy controls. Second, we demonstrate that three key cytokines, TNF, MCP-1 (CCL2), and IL1RA may be important in mediating this cytotoxicity. And third, RNA-seq of neutrophils from breast cancer patients and controls identified the gene *PRKCI* to be overexpressed in patient neutrophils relative to neutrophils from cancer-free individuals. Furthermore, TNF stimulated neutrophil cytotoxicity against breast cancer cell lines and induced *PRKCI* expression in the subset of samples that had low basal expression of this gene. This suggests the possibility that TNF-primed neutrophils may specifically inhibit metastasis to the lungs.

In 90 newly diagnosed breast cancer patients with no evidence of metastasis, and 64 women with no history of any cancer, we demonstrate a statistically significant difference in average cytotoxicity values. Among primary breast cancer patients, the highest neutrophil cytotoxicity was in the ER+/HER2− breast cancer patients, which is traditionally the most favorable subtype of breast cancer^9^ and that the average neutrophil cytotoxicity of ER−/ HER2+ patients was the lowest of all our samples, including controls, although sample sizes were too small to assess the significance of this result. Interestingly, in our samples, patients with DCIS alone had a higher neutrophil cytotoxicity than those patients with primary invasive breast cancers but no evidence of metastasis. Perhaps at this very early stage of disease, higher neutrophil cytotoxicity reflects an improved prognosis, suggesting that the immune system may protect against progression to invasive disease. However, the metastatic patient group, which presented with diffuse metastatic disease, often to visceral organs, had the highest neutrophil cytotoxicity. Previously published work by Granot *et al.* suggests that host cell factors and the micrometastatic niche are critical for inducing neutrophil cytotoxicity. Specifically, a microenvironment enriched for TGF-β can actually promote metastasis and downregulate neutrophil cytotoxicity.^6^ As such, the neutrophils from metastatic patients, while exhibiting high neutrophil cytotoxicity when the neutrophils are isolated and tested *ex vivo* in cell culture conditions, may have their cytotoxicity reduced *in situ* due to high levels of TGF-β present at metastatic sites. Alternatively, the role of neutrophil cytotoxicity could be outweighed by other tumor and immune host factors, particularly as the metastatic burden of disease increases.

To identify the tumor-secreted factors that may prime neutrophils, we analyzed cytokines in the serum of the patients and controls. In our pilot screen, we identified TNF, MCP-1 (CCL2), and IL1A as being increased in patients relative to controls. This initial result suggested that these cytokines may be involved in cytotoxicity as neutrophils isolated from breast cancer patients have higher cytotoxicity relative to neutrophils isolated from cancer-free controls. In a larger sample size, (54 patients and 35 cancer-free controls), we found IL1RA, MCP-1, and TNF were present at significantly higher levels in samples with high neutrophil cytotoxicity relative to samples with low neutrophil cytotoxicity. Furthermore, treating neutrophils from either patients or healthy controls with these cytokines significantly increased neutrophil cytotoxicity, suggesting that the elevated serum levels of these factors may be functionally important.

In an RNA-seq analysis of neutrophils isolated from breast cancer patients and healthy controls, we identified a small number of genes differentially expressed. One of these genes, *PRKCI* showed a positive correlation between its expression and cytotoxicity. *PRKCI* encodes a member of the ‘atypical’ protein kinase C subfamily. *PRKCI* has been described as an oncogene in non-small cell lung cancer (NSCLC), and was found overexpressed in non-small cell lung cancer cells and to play a role in cell transformation.^10,11^ The role of *PRKCI* in neutrophils has not been elucidated, however other protein kinase C family members have been implicated in assembly of the NADPH oxidase complex, which is required for neutrophil-mediated cell cytotoxicity by production of reactive oxygen species.^12^ In a study investigating the expression of protein kinase C family members in neutrophils, *PRKCI* expression was detected in terminally differentiated leukemic neutrophils.^13^ Interestingly we observed that treatment of human neutrophils with TNF induced *PRKCI* expression and cytotoxicity in a subset of samples in which basal levels of *PRKCI* were low. Further functional studies will be necessary to show that *PRKCI* directly contributes to neutrophil cytotoxicity.

Recent evidence suggests that tumor-infiltrating polymorphonuclear leukocytes may be associated with outcomes across many solid tumors,^14^ but that the quantity of neutrophils in the circulation may not influence outcomes. However, here we demonstrate that the key function of circulating neutrophils may not be related to exact quantities of neutrophils but rather whether circulating cytokines prime select neutrophils for cancer cell cytotoxicity, and in this way have a role in tumor surveillance. Another recent study by Wculek and Malanchi^15^ paradoxically reported a pro-metastatic role for neutrophils in the development of lung metastases, highlighting the ongoing controversy surrounding the role of neutrophils in tumor development and metastasis. This controversy may partially be explained by recent studies demonstrating a great deal of heterogeneity within neutrophil populations during tumor development.^16^ This high level of heterogeneity may also explain the paucity of commonly differentially expressed genes identified in our RNA-seq analyses. Further investigation of the various neutrophil subpopulations present during tumor development may aid in elucidating the pro- versus anti-tumorigenic functions of neutrophils.

Finally, although experimentally challenging, developing a model in which we can test the effect of cytokine-stimulated neutrophils on metastasis *in vivo* would be of particular interest to explore the therapeutic potential of our findings, and is currently being pursued. The finding that 2 h of cytokine stimulation was sufficient to stimulate neutrophil cytotoxicity in overnight cytotoxicity assays suggests that neutrophil transfer of stimulated neutrophils may be possible.

In conclusion, we demonstrate that circulating neutrophils from breast cancer patients have significantly increased anticancer cell cytotoxicity and that this activity can be stimulated by selected cytokines, namely TNF, which is elevated in breast cancer patients and associated with neutrophil cytotoxicity. These results provide early evidence that a better understanding of the nature of select neutrophils may facilitate the path towards novel immunotherapies in which the transfer of cytokine-stimulated neutrophils into breast cancer patients may help steer the function of neutrophils towards an effective immunologic blockade of disease progression and metastasis. As our understanding of the immune system continues to increase and thus elucidate critical tumor–microenvironment interactions, leveraging neutrophil function may be important in rendering metastatic niches less hospitable to cancer growth.

## Materials and methods

### Cell lines

The MDA-MB-231 mammary tumor cell line was purchased from ATCC (Manassas, VA, USA).

### Human blood samples and neutrophil isolation

Blood samples (~10 cc) were collected from healthy (*n*=64) controls or from patients (*n*=165) who were already having blood drawn for pre-surgical testing as part of a Memorial Sloan Kettering Cancer Center institutionally approved protocol. Controls had no history of any kind of cancer, denied recent illnesses or history of autoimmune disorders, and were not taking any immune-modulating or anti-inflammatory medications. Patients with newly diagnosed non-metastatic breast cancer and had no prior exposure to chemotherapy agents. Patients were consented accordingly as part of the approved protocol and had their blood drawn prior to definitive surgical removal (lumpectomy or mastectomy) of their primary tumor. Blood samples were transferred to the lab for neutrophil isolation no >1 h post blood draw. One milliliter of the heparinized blood was centrifuged for 5 min, and the serum (supernatant) was collected and stored at −80 °C until required for analysis. The remainder of the heparinized blood was mixed with an equal volume of Dextran 500 (3% in saline) and incubated for 30 min at room temperature. The resultant supernatant was layered on top of histopaque 1077 (Sigma, St Louis, MO, USA) and centrifuged. The neutrophil-containing pellet was resuspended in 10 ml 0.2% NaCl for 30 s to facilitate erythrocyte lysis. Isotonicity was restored by the addition of 10 ml 1.6% NaCl. The neutrophil pellet was then washed three times with Hank’s balanced salt solution and resuspended in RPMI containing 2% fetal bovine serum.

### *In vitro* cytotoxicity assays

Luciferase-labeled MDA-MB-231 cells were plated in a 96-well, flat bottom white polystyrene tissue culture plate (5000 cells per well) in RPMI 2% fetal bovine serum. Four hours later, purified neutrophils were added to the plated MDA-MB-231 cells (5000 cells per well) and co-cultured overnight (at least 6 replicates per sample). Following overnight co-culture, the wells were washed with PBS, cells lysed and luciferase activity, a readout of cell viability, was measured using the Clarity (Bio-Tek, Winooski, VT, USA) microplate luminescence reader and the Dual-luciferase Reporter Assay System (Promega, Madison, WI, USA).

In experiments involving cytokine stimulation of neutrophils, the cytokines (all from R&D (Minneapolis, MN, USA) and used at 100 ng/ml) were added to the culture immediately after neutrophils were added, or were incubated with the neutrophils in culture media for 2 h at 37 °C prior to the neutrophils being added to MDA-MB-231 for overnight co-culture (at least 6 replicates per sample per treatment group). In experiments where the neutrophils were incubated with the cytokines for 2 h, following the incubation period the neutrophils were centrifuged, cytokine-containing media aspirated, neutrophil pellet washed with PBS, and then the neutrophils were resuspended in fresh RPMI 2% and added to the plated MDA-MB-231 cells for overnight co-culture. Cytotoxicity was determined as described above. Experiments were repeated at least three times with similar results.

### Serum cytokine profiling

Serum profiling was performed using a Luminex Cytokine assay, a magnetic bead-based sandwich immunoassay for cytokines. Thirty-seven samples were assayed on the MILLIPLEX MAP Human Cytokine/Chemokine Magnetic bead panel (HCYTOMAG-60K, 38-plex premixed beads) (EMD-Millipore, Jaffrey, NH, USA) and 144 samples were assayed on custom arrays that assay for MCP-1, TNFα, IL1A, and IL1RA (Cat. # HCYTOMAG-60K, HCYTOMAG-60K-PX29, HCYTOMAG-60K-PX30, HCYTOMAG-60K-PX38, HCYTOMAG-60K-PX41 (EMD-Millipore) according to the manufacturer’s instruction. Undiluted serum samples were analyzed in duplicate wells by Luminex FlexMap 3D (Luminex, Austin, TX, USA). Cytokine concentrations were determined by Luminex Xponent 4.2 using 5-p log analysis.

### RNA-seq

RNA samples (*n*=60) for RNA-seq were extracted with Trizol reagent, according to manufacturer’s protocol by the MSKCC Genomics Core facility. RNA-seq was performed at the New York Genome Center. RNA quality control was done on Agilent BioAnalyzer (Agilent Technologies, Santa Clara, CA, USA), and Qubit was used for quantity measurement. RNAseq libraries for the HiSeq 2500 were prepared using the Illumina TruSeq Stranded Total RNA Preparation kit. Briefly, 100 ng of total RNA is purified by Ribo-Zero (Illumina, Inc., Hayward, CA, USA) to remove rRNA and fragmented by divalent cations under elevated temperature. The fragmented RNA undergoes first-strand synthesis using reverse transcriptase and random primers. Second strand synthesis creates the complementary DNA (cDNA) fragments using DNA polymerase I and RNaseH. The cDNA fragments then go through end repair, adenylation of the 3′ ends, and ligation of adapters. The cDNA library is enriched using 10 cycles of PCR and purified. Quality control consisted of assaying the final library size using the Agilent BioAnalyzer (Agilent Technologies, Santa Clara, CA, USA) and quantifying the final library by quantitative PCR and PicoGreen methods. Sequencing was performed on the HiSeq 2500 instrument using v3 chemistry with 2×50 bp reads. Sequencing libraries were loaded onto the HiSeq 2500 flowcell for clustering on the cBot using the instrument specific clustering protocol. The HiSeq 2500 is capable of generating 180-M passed filter 2×50 bp sequencing reads per flow cell lane. Samples were multiplexed in order to obtain a minimum of 60 M PF reads per sample. The RNA-Seq reads were aligned to the human reference sequence hg19 with the RNASeq aligner STAR (version_2.3.1x). Genes annotated in Gencode version 18 were quantified with featureCounts (subhead package version 1.4.3-p1).

### qRT-PCR analysis

RNA was extracted using the RNeasy kit (Qiagen, Valencia, CA, USA) and cDNA was generated from 1 ug of RNA using SuperScript III First-Strand Synthesis System (Invitrogen, Grand Island, NY, USA). Quantitative PCR was performed on 8 samples using SYBR Green QuantiTect Primer Assay (Qiagen) according to the manufacturer’s instructions in a 7900HT Fast-Real Time PCR System Instrument (Applied Biosystems, Grand Island, NY, USA). Primer pairs for the individual genes were obtained from the bioinformatically validated QuantiTect Library and are as follows: *PRKCI* (QT00058954) and *CPT1A* (QT00082236). The fold changes in gene expression were calculated using the delta–delta CT method (assays were performed in triplicate).

### Statistical analyses

The analyses performed were of two types based on the association being evaluated. The first type was used for cytotoxicity as numeric variable. We tested the association between cytotoxicity and either cytokine levels (pg/ml) or gene expression (normalized read counts) using Spearman's rank correlation; the *P*-value represents the level of significance for the null hypothesis that the correlation is zero. The second type of analysis was used for comparing cytokine levels or gene expression across two or more groups. The grouping variable is tumor status (patients versus controls) or cytotoxicity categorized into high and low levels. For the two-group comparison Wilcoxon–Mann–Whitney rank sum test is used and for more than two groups (controls, high patients, and low patients) the Kruskal–Wallis test is used. For gene expression analysis 57,445 transcripts were quantified with many of them having zero read counts for a large number of samples. Thus, a filter of read count of at least 5 for >75% of transcripts was applied (only 16,410 transcripts pass the filter). Benjamini–Hochberg false discovery rate correction was used to adjust for multiple testing. For statistical analysis of cytotoxicity and qRT-PCR assays, the data is presented as mean±s.e.m. and was analyzed using a two-sided Student’s *t-*test with *P*<0.05 indicating significant differences.

## Figures and Tables

**Table 1:** Mean neutrophil cytotoxicity in invasive and metastatatic breast cancer patients by hormone status

	*ER+/HER2− (% Cytotoxicity (*n*))*	*ER−/HER2−(% Cytotoxicity (*n*))*	*ER+/HER2+ (% Cytotoxicity (*n*))*	*ER−/HER2+ (% Cytotoxicity (*n*))*
Invasive	14.4 (59)	13.8 (15)	11.4 (7)	9.0 (7)
Metastatic	18.0 (38)	17.4 (6)	20.3 (5)	21.6 (7)

Abbreviations: ER, estrogen receptor; HER2, human epidermal growth factor receptor 2.
