# Supplementary material for: TNF is a key cytokine mediating neutrophil cytotoxic activity in breast cancer patients
Source: NPJ Breast Cancer. 2016 Apr 20;2:16009–. doi: 10.1038/npjbcancer.2016.9 (PMC5515342; doi:10.1038/npjbcancer.2016.9)
Supplement: Supplementary Figures [file npjbcancer20169-s1.ppt]

## Slide 1
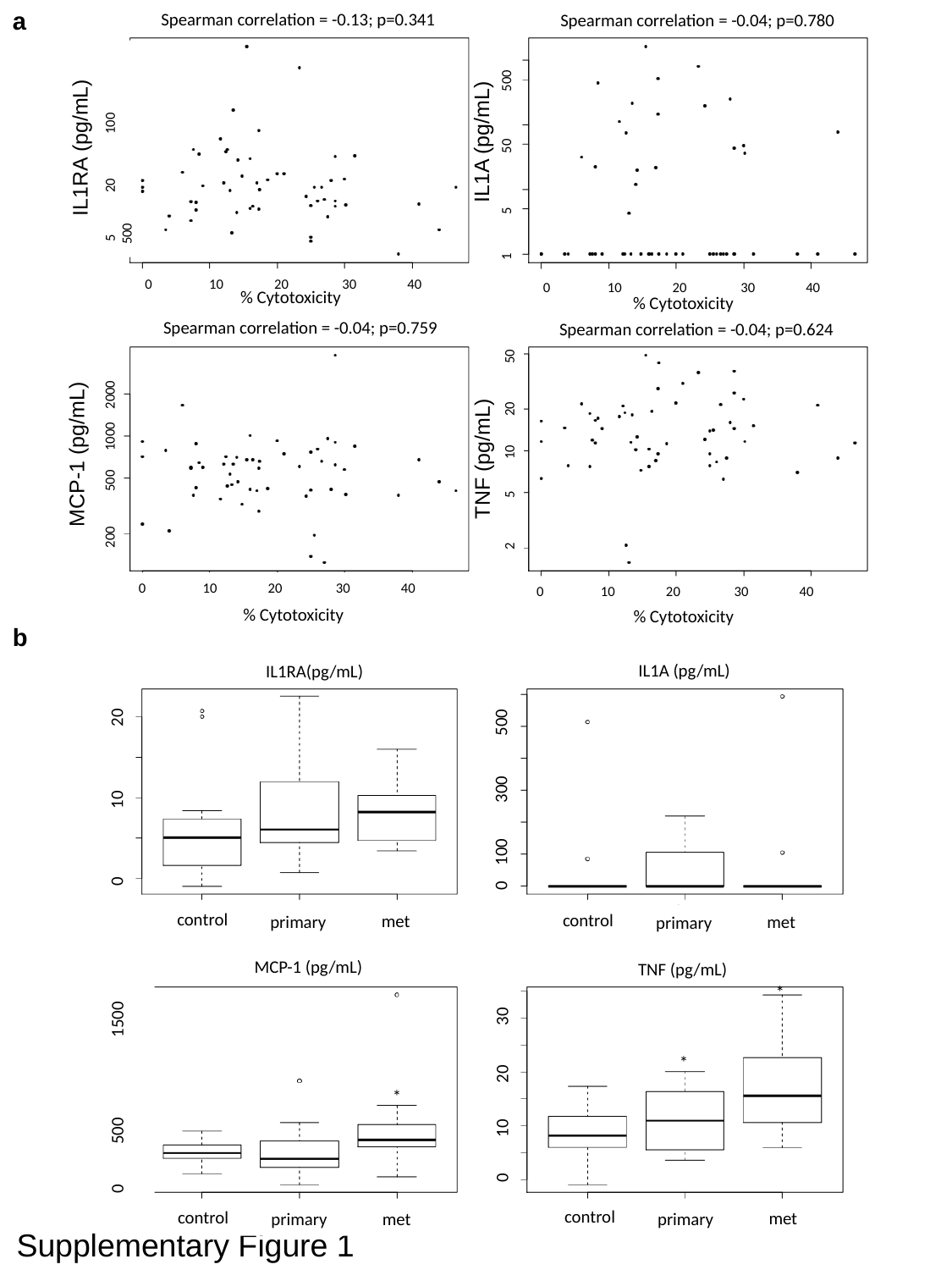

a
Spearman correlation = -0.13; p=0.341
Spearman correlation = -0.04; p=0.780
IL1A (pg/mL)
 5 20 100 500
IL1RA (pg/mL)
 1 5 50 500
0 10 20 30 40
0 10 20 30 40
% Cytotoxicity
% Cytotoxicity
Spearman correlation = -0.04; p=0.759
Spearman correlation = -0.04; p=0.624
 2 5 10 20 50
MCP-1 (pg/mL)
TNF (pg/mL)
 200 500 1000 2000
0 10 20 30 40
0 10 20 30 40
% Cytotoxicity
% Cytotoxicity
b
IL1A (pg/mL)
IL1RA(pg/mL)
0 100 300 500
0 10 20
control
control
met
primary
met
primary
MCP-1 (pg/mL)
TNF (pg/mL)
*
*
0 10 20 30
0 500 1500
*
control
control
met
met
primary
primary
Supplementary Figure 1
